# Opportunities and Concerns of Gamified, Extended Reality for Home-Based Motor Rehabilitation for Children With Brain Injury: Qualitative Case Study on Design Elements Related to the Engagement and Fatigue Perspectives

**DOI:** 10.2196/84013

**Published:** 2026-06-02

**Authors:** Eivind Kolstad, Nenad Pavel, Alexis Ken Sosmena Cartajenas, Trust Saidi, Ingvild Kristina Hurum Rosseland, Åse Bergheim, Nora Synnøve Mørk, Kathinka Granum Selmer-Olsen, Parisa Gazerani, Shefaly Shorey, Minna Pikkarainen

**Affiliations:** 1 Department of Product Design Technology Art and Design Oslomet – Oslo Metropolitan University Oslo, Oslo Norway; 2 Oslo Municipality Oslo, Oslo Norway; 3 Alice Lee Centre for Nursing Studies, Yong Loo Lin School of Medicine National University of Singapore Singapore, Singapore Singapore

**Keywords:** extended reality, brain injury, rehabilitation, engagement, fatigue

## Abstract

**Background:**

Acquired brain injuries are injuries that occur after birth and are a leading cause of long-term disability and death in children and young adults. They may result from trauma, hypoxia, stroke, infection, or a variety of other causes. Fatigue is one of the most common and underrecognized consequences of pediatric acquired brain injury, often expressed behaviorally rather than verbally. Traditional rehabilitation programs are frequently static and cognitively demanding, limiting engagement and therapeutic outcomes. Extended reality (XR) technologies offer new opportunities to address these challenges by enabling interactive, adaptive, and motivating home therapy environments. However, few XR systems are co-developed with children and therapists, and there is limited knowledge about how to co-design engaging, gamified, XR-based motor rehabilitation solutions that take into account children’s fatigue.

**Objective:**

This study explores why specific gamification and XR design elements facilitate or hinder engagement and effective fatigue response during rehabilitation for children with acquired brain injuries.

**Methods:**

A qualitative case study approach was employed with a total of 25 participants (22 provided consent), combining co-design workshops, interviews with health care professionals, observational data, and iterative user testing with children ages 8-16, their parents, and the clinical team. Participants who provided consent included 4 children with acquired brain injury (ages 8-16), 4 parents, 4 clinicians, and 10 healthy children involved in early ideation only. The XR prototype was developed using Unity, Cognitive3D, and the Meta Quest 3 headset. Engagement and fatigue related to prototype use were evaluated using subjective measures adapted from the User Engagement Scale and the Virtual and Mixed Reality Fatigue Scale, supplemented by thematic analysis of interview and workshop data.

**Results:**

Children demonstrated higher engagement with short, modular XR sessions (3-10 minutes) that included interactive game elements and preserved visibility of their surroundings. Fatigue was identified through behavioral cues such as gaze, posture, and responsiveness. Therapists emphasized the importance of adaptive difficulty, personalization, and simplified environments. A therapist-facing dashboard was developed to visualize behavioral fatigue-related cues, consistent with clinician observation.

**Conclusions:**

This qualitative case study provides preliminary insights into how XR-based rehabilitation, when co-designed with children and clinicians, may support engagement and facilitate observation of fatigue-related behaviors in pediatric brain injury contexts. The findings provide design-oriented insights for creating engaging XR home-rehabilitation experiences while accounting for fatigue-related limitations (eg, short, modular sessions; visual grounding; adaptable challenge). The results of our study indicate a need for objective fatigue measurement within XR solutions to adjust content in ways that support both engagement and fatigue considerations. However, fatigue detection was not validated in this study and should be addressed in future research.

## Introduction

Pediatric acquired brain injury (ABI) is a leading cause of long-term disability among children and adolescents, affecting cognitive, motor, sensory, and emotional domains [1,2]. In Norway alone, thousands of children experience such injuries annually due to trauma, illness, or medical conditions [3]. Data from the Oslo region show that approximately 29 out of every 100,000 children are hospitalized each year due to traumatic brain injuries of varying severity [4]. ABIs are insults that occur after birth and are a leading cause of long-term disability in children and young adults. They stem from trauma, hypoxia, stroke, infection, and other factors, producing varied deficits in cognition, behavior, metabolism, motor control, perceptual-motor skills, and sensation. Many ABIs cause hemiplegia—one-sided weakness or paralysis—compromising upper-limb function [5]. Typical treatment options for (physical) pediatric ABI include standard physical therapy, occupational therapy, and robot-assisted training. Over extended treatment periods, however, many children with ABI may struggle to engage or may discontinue therapy [6].

It has been reported that insufficient motivation is a major barrier to children with ABI reaching their functional potential in rehabilitation [7]. Thus, to improve adherence, more engaging and enjoyable approaches are needed [6]. Exoskeleton-based therapy supports high-repetition, high-intensity rehabilitation practices. When paired with interactive environments such as extended reality (XR), it can increase motivation and functional relevance, with reported gains, for example, in hand and finger coordination in children with cerebral palsy [5].

Emerging research suggests that XR, an umbrella term for virtual reality (VR), augmented reality (AR), and mixed reality, holds promise in addressing challenges in pediatric rehabilitation. XR tools can simulate interactive, playful, and personalized environments that increase therapy engagement, particularly through gamification [8]. Although there is not yet much research evidence on the effectiveness of VR-based pediatric ABI programs [9], and even less on AR-based programs in home settings [10], existing studies have shown that XR can support motor function recovery, improve attention, and sustain motivation when integrated into goal-directed therapy [11,12]. VR has emerged as a promising and relatively effective approach for rehabilitation of pediatric ABI, with the potential to improve functional status, physical activity, balance, and cognitive and motor skills [6,13-15], whereas AR has been shown to have positive effects on pediatric balance [10]. In home environments in particular, goal-directed VR programs, as a supplement to limited clinic therapy, have been shown to improve physical outcomes in children with ABI by enabling repetitive, intensive practice and sensorimotor feedback to boost motivation and motor learning [9].

Among the most prevalent and debilitating, yet often invisible, symptoms of pediatric ABI is fatigue, which can manifest cognitively, physically, or emotionally and significantly impact a child’s ability to engage in rehabilitation, education, and social activities [16]. Despite its prevalence, fatigue remains underreported and challenging to address in clinical contexts, especially among younger children who may lack the awareness or language to express their limits [17,18]. Clinicians and therapists often rely on indirect behavioral cues, such as reduced eye contact, changes in body posture, loss of focus, or disengagement, to identify exhaustion [19]. If not recognized and addressed, fatigue can lead to frustration, dropout from therapy, and diminished rehabilitation outcomes [20]. Concurrently, traditional rehabilitation programs are frequently static and repetitive, failing to meet children’s motivational and cognitive needs. This mismatch contributes to low adherence and an increased risk of long-term functional challenges [20]. While Mazzone et al [21] articulate the complexity of fatigue assessment and management in pediatric ABI rehabilitation, our study aims to address a critical gap by demonstrating how XR design can operationalize these clinical processes through fatigue-sensitive interaction, adaptive engagement, and observable behavioral indicators.

Current XR-based rehabilitation solutions for pediatric populations are not designed to respond to fatigue, and it remains unclear how such solutions should be designed to account for the fatigue perspective. Besides, they do not sufficiently consider individual differences in energy levels, motivation, or cognitive capacity [22]. For example, a recent systematic review and meta-analysis examining cognitive fatigue in children following ABI found that nearly three-quarters of impacted individuals (74.6%) experience ongoing cognitive fatigue [23]. Despite its widespread occurrence, the study identified a lack of standardized, evidence-based interventions specifically designed to address this issue within pediatric rehabilitation settings, highlighting the inadequacy of current tools in recognizing and responding to fatigue. There is also limited research on integrating engagement theory with fatigue in the context of XR-based health care design [24], as well as a limited understanding of intuitive interfaces that bridge technical data with clinical applications [25]. Finally, only a few XR rehabilitation solutions are co-designed with children, therapists, and caregivers, limiting their usability, accessibility, and clinical relevance [26].

Thus, there is a lack of knowledge about how to engage and motivate children with pediatric ABI during interactive XR-based motor rehabilitation tasks, and how to design engaging XR-based motor rehabilitation solutions for ABI that take children’s fatigue into account.

To address these gaps, this qualitative case study explores which gamification and XR design elements facilitate or hinder engagement in home-based motor rehabilitation for children with ABI, and how fatigue-related considerations shape design requirements and session structure. This is examined through the following subquestions: (1) How do the clinical teams perceive opportunities and concerns? (2) How are fatigue and engagement perceived by children and clinical teams during XR sessions? (3) How should XR solution design elements be adapted to preserve engagement while accounting for fatigue?

This was conducted as a qualitative case study [27] involving a total of 25 participants (22 provided consent), including healthy children (to help ideate the initial concept), members of the clinical team, children with ABI, and their parents in co-design workshops and individual interviews.

## Methods

### Research Design Overview

This study adopts a qualitative case study approach to explore how XR technologies can support the detection of fatigue-related symptoms in children with brain injuries. As defined by Yin [27], case studies investigate contemporary phenomena in real-life contexts, especially when the boundaries between the phenomenon and the context are not clearly evident. This method is well-suited for addressing “how” and “why” questions, such as how specific gamification and XR design elements facilitate or hinder engagement and effective fatigue response during at-home rehabilitation for a child with a brain injury. A single qualitative case study was selected because it enables a deep, contextual understanding of how a child’s engagement with XR motor rehabilitation fluctuates with fatigue, and what type of content supports engagement during use of the solution. Direct feedback from children immediately after XR solution trial sessions, along with observations and discussions with clinical teams and parents, provided rich data to enhance understanding of the phenomena related to the research question. Co-design with the child, parents, and the clinical team translated these insights into tailored, engaging content and fatigue-aware adaptations (eg, dynamic difficulty adjustment, micro-breaks, and session pacing) to optimize usability and adherence. The prototype was developed as a motor rehabilitation support tool intended to increase engagement and provide a structured, safe, home-like training experience. Although fatigue detection was discussed during the workshops, this study did not aim to validate automated fatigue detection. Instead, fatigue was treated as a design constraint, explored through stakeholder feedback and clinician-observed behaviors during short sessions.

**Figure 1:**
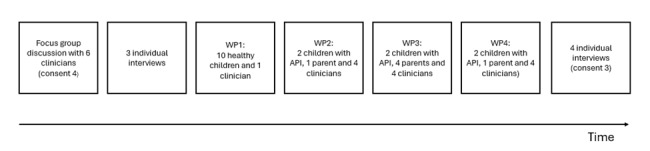
Timeline illustrating the sequence of data collection activities.

The case centers on co-developing an XR rehabilitation solution with clinicians (eg, physical and occupational therapists), children with ABIs, and their parents. Data collection involved 25 participants, including members of the clinical rehabilitation team, children with ABI, and their parents (Figure 1). Multiple data sources—including workshops, interviews, observations, and the Engagement Scale and Virtual and Mixed Reality Fatigue Scale (VMRFS) surveys (see Multimedia Appendix 1)—provided deep insights into user needs and therapeutic impact. Following Yin’s [27] principles—clear case boundaries, multiple sources of evidence, and a maintained chain of evidence—the study used an iterative, reflective process to inform design decisions, ensuring that the solution is engaging, clinically grounded, and responsive to real-world needs. The study results were reported in accordance with Journal Article Reporting Standards [28].

### Researcher Description

The research team combined complementary expertise: EK led data collection and analysis, supported by NP’s contributions to study design and workshop facilitation, and AKSC’s assistance with data collection. Clinical partners IKHR, ÅB, NSM, and KGSO contributed to patient recruitment, provided clinical insight, and reviewed the manuscript. Senior academics—PG, SS, and the lead supervisor, MP—strengthened the methodological and scientific quality of the study, with MP overseeing study conception, ethics, project coordination, and consolidation of the manuscript for publication.

### Study Participants, Participant Recruitment, and Data Sources

Participants were recruited through collaboration with the Norwegian Municipality, Norwegian hospitals, and researcher networks (healthy children) using purposive sampling [29]. XR generally benefits from age-appropriate design; however, in pediatric brain injury rehabilitation, adaptation based on functional capacity and fatigue was prioritized over chronological age in this study. Purposive sampling was used to recruit participants who could provide information-rich insights relevant to the study’s aims, specifically (1) children with ABI who represented typical rehabilitation cases in terms of functional variability and fatigue patterns; (2) clinicians with direct experience treating such children; and (3) healthy children who could contribute to early-stage usability and interaction design. This approach ensured diversity in functional abilities, therapeutic roles, and user perspectives, rather than diversity in demographic characteristics such as chronological age.

A description of participant recruitment is provided in the participant flow diagram (see Multimedia Appendix 2). Overall, 25 individuals—healthy children, clinicians, and children with ABI—were contacted about the study. In total, 22 participants provided consented data: 10 healthy children (ideation phase), 4 children with ABI, 4 parents, and 4 clinicians. Data collection proceeded in 3 stages: (1) an early ideation workshop with healthy children and initial clinician interviews; (2) iterative co-design and playtesting workshops with children with ABI, parents, and clinicians; and (3) follow-up clinician interviews after the final iteration.

The first phase of the study included a workshop with 10 healthy children aged 9-16, followed by a meeting with 6 clinicians and individual interviews with 3 clinicians who had hands-on expertise in the rehabilitation of children with ABI. The first workshop was conducted as part of the OsloMet Design for Experience course. This workshop aimed to gain a general understanding of preferences for rehabilitation games among children in this age group. In total, 10 healthy children aged 9-16 participated in the workshop. Six different design student groups (4-6 students in each group) created paper prototypes of potential rehabilitation games. The children were asked to evaluate these early concepts and provide open feedback. During the sessions, the research team conducted observations and took notes on the children’s excitement and reactions to the different design concepts. These observations served as a baseline for selecting 3 initial design concepts for further discussion with the clinical team.

The next step in the research process was to conduct a focus group discussion with clinicians working with children with ABI to better understand the rehabilitation status and needs of this population, as well as to gather feedback on their preferences regarding the preliminary design ideas and generate initial concepts for the XR solution. However, only 4 of the 6 clinicians who participated in the focus group discussion and 2 of the 3 clinicians who participated in the individual interviews provided consent for data usage. Consequently, only the consented data were included in the formal analysis. Data from the remaining 4 clinicians were used as background input to inform the development of the initial XR design concepts, but were not included in the analyzed dataset.

The next phase of the study (4 workshops) included 4 children with ABI (3 participated with their parents and 1 with an assistant), aged 9-16, and clinicians (including occupational and physical therapists) with rehabilitation experience. The focused objective of the workshops was to explore stakeholder perceptions (specifically concerns and opportunities related to XR-based rehabilitation), understand how fatigue and engagement were experienced during and after XR sessions, and identify how the XR solution should be adapted to preserve engagement while accounting for fatigue. In this study, fatigue-related cues referred to clinician-observed, naturally occurring behavioral changes during interaction, such as reduced responsiveness, slowed reaction time, reduced movement amplitude, changes in posture, gaze disengagement, or an increased need for prompts. These cues were used to inform interpretation of engagement and to guide fatigue-aware design decisions (eg, session length, breaks, and sensory load). Observations were documented through field notes, workshop video review, and clinician reflections. Fatigue was not induced; sessions were kept short and supervised by the clinical team, and activities were paused if clinicians or caregivers determined that the child needed a break. No specialized technical training was required to identify these cues; however, clinicians’ familiarity with pediatric ABI strengthened the contextual interpretation.

The solution was intended to be engaging while supporting motor rehabilitation and facilitating responses to fatigue-related symptoms in children with brain injuries. After the fourth workshop, 3 additional individual interviews were conducted with the same clinical team members who had participated in all workshops. The purpose of these interviews was to gain a deeper understanding of their perspectives on opportunities and concerns, fatigue and engagement during XR sessions, and XR design elements needed to preserve treatment goals while accounting for fatigue.

The study prioritized the collection of actionable insights to guide iterative refinement of XR prototypes rather than aiming for exhaustive thematic saturation. Accordingly, a small, purposefully selected sample was considered methodologically appropriate. Clinicians were selected based on their direct practical experience in pediatric ABI rehabilitation, including occupational and physical therapists and a pediatric neurologist involved in home- and outpatient-based care. Children with ABI were purposively selected to reflect diversity in age (8-16 years), type and cause of injury (eg, stroke, traumatic brain injury), functional abilities, and observed fatigue characteristics, while still being able to safely participate in XR-supported motor activities. Parents were included, when appropriate, to provide complementary perspectives on fatigue and engagement in the home rehabilitation context. In the early ideation phase, healthy children were intentionally recruited as proxy users to explore general gameplay preferences and interaction concepts before involving children with ABI, thereby reducing participant burden and informing initial design decisions. This sampling strategy supported the study’s iterative co-design process and the development of fatigue-sensitive XR design insights.

The approach aligns with rapid prototyping methodologies, which emphasize the value of targeted feedback to inform design decisions rather than comprehensive theme development [30]. The study also adhered to participatory research principles by engaging a representative sample of end users, including clinicians whose perspectives are critical to the intervention’s relevance and usability. Furthermore, the total sample size (10 healthy children, 4 children with ABI, 4 parents, and 7 clinicians, comprising a total of 25 participants) falls within the range recommended by Creswell and Miller [31] for phenomenological inquiry, which suggests between 5 and 25 participants. Data collection involved workshops, interviews, observations, and surveys to support triangulation and ensure rich, contextual insight into the design, use, and evaluation of the XR rehabilitation prototype, as shown in Table 1.

**Table 1:** Overview of data types, sources, and analytic uses in a mixed methods co‑design study developing and evaluating an extended reality therapeutic prototype for pediatric rehabilitation.

Data type	Sources	Use in the study	Quality^a^
Therapist/clinician interviews	Interviews	Identify clinical challenges, design needs, and real-world context	+++
Observations	Workshops	Capture behavior, verbal cues, and group dynamics during co-design and testing	++
Field notes	Workshops	Supplement observations with informal insights, environmental context, and tone	++
Feedback from patients	Questionnaires	Validate design usability and comfort; gather children’s preferences	+++
Feedback sessions with the clinical team	Interviews/workshops	Insight to build the prototype further	+++
Virtual and Mixed Reality Fatigue Scale	Postsession questionnaires	Assess physical, cognitive, and visual fatigue after extended reality use	+++
User Engagement Scale	Postsession questionnaires	Measure perceived engagement: attention, usability, and reward	+++
Prototype interaction videos	Workshop video recordings	Analyze interaction patterns, visual behavior, and fatigue indicators	++
Feedback sessions	Interviews/workshops	Collect insights from therapists on prototype refinement and clinical relevance	+++

^a^“Quality” indicates the research team’s perceived utility of each data source for answering the research questions (+++ high; ++ moderate), based on triangulation potential and richness of the data.

Semistructured interviews were conducted with 5 clinicians—physical therapists, occupational therapists, and a pediatric neurologist—experienced in pediatric brain injury rehabilitation. These clinicians provided insights into the behavioral presentation of fatigue, motivational challenges, and design features to enhance engagement and outcomes. The interviews (guide in Multimedia Appendix 3), lasting 45-90 minutes, followed a structured protocol covering rehabilitation practices, XR technology, and digital tools. Audio recordings were transcribed and thematically analyzed to inform XR prototype design. Co-design workshops were conducted with 2 key user groups: health care professionals and children with ABIs (or, in the first workshop, healthy children as proxies), as shown in Table 2, which summarizes their profiles, including age, diagnosis, and session involvement. “Quality” indicates the research team’s perceived utility of each data source for answering the research questions (+++ high; ++ moderate), based on triangulation potential and richness of the data.

**Table 2:** Overview of child and parent participants involved in the co‑design workshops, detailing each child’s type of brain injury, age, and participation across workshop sessions.

Child ID	Type of brain injury	Age (years)	Workshop participation
Child 1	None	10	1, 3, and 4
Child 2	While born	8	3 and 4
Child 3	Stroke	16	2 and 3
Child 4	Traumatic brain injury at a young age	11	2 and 4

The workshops had dual purposes: to collaboratively generate design ideas (early phase) and to collect feedback on different versions of the XR prototype. Co-design principles guided the development of the XR prototype through iterative workshops with children, parents, and therapists (see Table 3). Their contributions informed key design decisions, such as the inclusion of adaptive challenge mechanics, consideration of fatigue situations, natural rest prompts, and personalized rewards based on user interests.

**Table 3:** Overview of participant groups involved in each co-design workshop, summarizing the number of researchers, children with brain injuries (and children without injuries for workshop 1), parents, and clinicians who provided consent.

Stakeholder	Workshop 1	Workshop 2	Workshop 3	Workshop 4
Researchers	4	4	4	4
Children with brain injury	0 (10 children without brain injuries)	2	2	2
Parents	0	1	4	1
Clinicians		4	4	4

Each workshop session lasted approximately 120-180 minutes and included guided playtesting with the prototype, participant feedback through discussions and questionnaires, and follow-up discussions with the clinical team. The sessions were audio- and video-recorded, and postsession feedback was also documented through informal discussions and field notes. These workshops enabled iterative integration of feedback and supported the co-creation of features addressing user fatigue, safety, and engagement. Four children with varying types of ABIs participated across different workshops. Observations captured user interaction, task navigation, responses to feedback, and fatigue-related behaviors (eg, posture changes, slowed reaction time, disengagement). A structured protocol guided interviews conducted with the children after each session, supporting triangulation with other data sources. Participants who took part in the interviews and provided consent are shown in Table 4. To assess engagement and fatigue, 2 instruments were used: a simplified User Engagement Scale (UES) [32] and an adapted VMRFS [33]. The UES measured attention, usability, aesthetics, and reward, adjusted for pediatric cognitive levels (see Multimedia Appendix 1 for the scales used). The VMRFS assessed visual discomfort, cognitive strain, and physical fatigue. Both instruments were used qualitatively: each child with ABI was narratively guided through rating the prototype using the UES and VMRFS immediately after the session. These discussions were audio-recorded, transcribed, and analyzed alongside other qualitative data to identify patterns in motivation, usability, and fatigue. The instruments were administered in a simplified, interview-supported format appropriate for children. Items were used to prompt reflection on comfort, strain, and engagement and were analyzed qualitatively alongside interviews and observations, rather than as standalone quantitative outcome measures.

**Table 4:** Overview of interview participants summarizing their professional roles and duration of their individual interviews, conducted with informed consent.

Interviewed person	Role	Duration of interview (minutes)
Interviewee 1	Occupational therapist	50
Interviewee 2	Physiotherapist	60
Interviewee 3	Pediatric neurologist	50

### Data Analysis and Methodological Integrity

Data analysis followed Braun and Clarke’s [34] 6-phase thematic analysis framework. The process began with familiarization through transcription and repeated readings of the data. Initial codes were generated to capture key features related to fatigue, motivation, and engagement. These codes were then organized into broader themes, which were reviewed and refined to ensure coherence and accuracy. The final phase involved producing the report, supported by illustrative quotes and linked to relevant theoretical frameworks, including the UES [32] and Cognitive Load Theory [35]. Analysis was conducted first for the individual interviews and then iteratively extended to the workshop data. Given the small sample size and the risk of deductive identification, quotes are attributed by stakeholder group only.

This study ensured methodological integrity by drawing on diverse data sources—including interviews, co-design workshops, observations, interaction videos, and postsession engagement and fatigue scales—that captured the perspectives of children with ABI, parents, clinicians, and healthy children relevant to the study aims. Researcher influence was managed through reflexive practices such as memo writing, field notes, and supervisory discussions, and interviews were conducted using open-ended prompts to minimize bias. Reflective thematic analysis grounded the findings in the data, supported by direct quotes, behavioral descriptions, and triangulation across sources. Apparent contradictions—such as low self-reported fatigue despite observable fatigue cues—were integrated into the analysis rather than treated as inconsistencies. Contextual details about participants and settings enhanced transparency, and iterative prototype refinement functioned as a form of member validation, demonstrating the practical utility of the findings. Analytic consistency was maintained through collaborative coding and discussion, supported by reflexive notes and repeated engagement with the data. This approach ensured that the claims are well-supported, meaningful, and relevant to the design of fatigue-sensitive XR rehabilitation tools for pediatric ABI.

### Ethical Considerations

Approval from the Regional Committee for Medical and Health Research Ethics in Norway was deemed unnecessary because the study focused on health service research and therefore did not fall under the Health Research Act § 2. Instead, approval was obtained from the Norwegian Agency for Shared Services in Education and Research (SIKT; reference number 871403) to ensure data protection and privacy compliance. Participants received both oral and written information about the study, and informed consent was obtained before conducting the workshops and individual interviews. To protect participant privacy, all data were deidentified through the use of pseudonyms. Given the vulnerability of the study population, fatigue was not deliberately induced. Observation of fatigue cues was limited to naturally occurring behaviors during short, supervised XR sessions conducted in collaboration with the experienced clinical rehabilitation team.

All participants—children, parents/guardians, clinicians, and healthy children involved in the early ideation phase—received written and oral information about the study, and parents/guardians provided informed consent before participation. Participants received gift cards as compensation for their efforts. Study data were deidentified using pseudonyms, and all audio files, transcripts, and workshop materials were securely stored on OsloMet’s research server with access restricted to the research team, ensuring confidentiality and compliance with GDPR (General Data Protection Regulation). No identifiable images or videos of participants are included in the manuscript or its multimedia appendices.

## Results

### Themes Identified

The data analysis revealed 4 main themes: fatigue detection challenges; engagement and motivation; adaptive therapy design; and clinical utility, therapist needs, and child-centered co-design. These themes were further divided into 16 subcategories (Multimedia Appendix 4).

### Clinical Team Concerns and Opportunities for XR-Based Home Rehabilitation of Children With API (Based on Individual and Focus Group Interviews)

#### Fatigue As a Central Barrier to Rehabilitation

All clinicians unanimously emphasized fatigue—both cognitive and physical—as a pervasive yet often underrecognized barrier in therapy:

Children rarely verbalize tiredness directly; instead, signs emerge behaviorally through irritability, lack of engagement, shortened attention spans, or reduced physical coordination.

Clinicians noted that fatigue often masquerades as disinterest, laziness, or poor behavior, making it difficult to detect without strong contextual knowledge of the child. As one clinician explained, “Children may try to keep up even when they don’t have the capacity for it, which can be mistaken for behavioral problems.” Another therapist noted, “Children pull the elastic further than adults would. They often don’t know their limits and just try to keep going,” highlighting the need for therapists to interpret behavioral cues rather than rely solely on self-report.

#### The Engagement-Fatigue Paradox

When designing AR solutions, it is challenging to determine the appropriate balance between fatigue and engagement. One therapist described the delicate balance between engaging a child through stimulation and avoiding overstimulation that may lead to rapid fatigue or frustration: “You have to push a little, but not too much. You have to dose correctly.” A physical therapist emphasized that engaging therapy is critical for maintaining motivation; however, cognitive and physical overload can cause children to shut down. Clinicians highlighted the importance of short, adaptive sessions tailored to the child’s energy level and day-to-day condition. One therapist described this dynamic as a “balancing loop,” in which excessive stimulation leads to exhaustion, reducing engagement and, consequently, limiting therapeutic effectiveness.

#### Motivation Through Personalization and Play

Motivational breakdown was described as a key reason children disengage from therapy, particularly after setbacks at school or in social life. Clinicians stressed the importance of making therapy fun, meaningful, and personalized. One physical therapist noted, “Children are more likely to engage when they understand the purpose of the activity and can experience mastery.” Games that incorporate levels, points, rewards, or themes aligned with children’s interests (eg, dinosaurs, magic, sports) were viewed as highly beneficial. “They train better when they forget they are training,” one therapist observed, underscoring the value of gamified experiences. Another therapist added, “If it’s too challenging, they fall off quickly. It has to be something they feel they can master.”

#### The Role of Adaptive XR Technologies

All clinicians were optimistic about the potential of XR—particularly AR—to support motivation and extend therapy beyond clinical hours. “AR lets them stay aware of their surroundings, which is really important when balance and orientation are affected,” one therapist explained. AR was preferred over VR because of its ability to preserve spatial awareness and minimize safety risks for children with balance or visual impairments. Clinicians envisioned XR as a “tool in the toolbox,” ideally used to supplement traditional therapy with personalized, game-like activities that adapt in difficulty and provide feedback on progress. Objective data generated through XR (eg, movement patterns, reaction time, engagement levels) were viewed as promising for tracking fatigue, tailoring exercises, and evaluating progress over time:

It would be very helpful to get data that shows change, like how much they’re using one arm compared to the other.

#### Clinical and Ethical Considerations

While enthusiasm for XR was high, several practical concerns were raised. These included overstimulation, the risk of demotivation if exercises are too difficult or too easy, and the importance of involving both therapists and families in guiding the child’s use of XR tools. “The content must be adapted and user-friendly, not just for the child, but also for therapists and parents,” one therapist emphasized. Safety, particularly in VR environments, was a recurring concern. “If you take away their vision, and they already have balance or sensory problems, it can be really dangerous,” warned one physical therapist. Clinicians stressed that XR systems must be adaptable, intuitive, and grounded in therapeutic intent—not merely another game. Together, these insights highlight the need for rehabilitation technologies that are adaptable, engaging, and clinically grounded. The interviews informed both technical priorities (eg, fatigue monitoring and difficulty scaling) and experiential aspects (eg, personalization, flow states, and emotional safety) that are important for XR system development. As one therapist put it, “It needs to be motivating and challenging enough, but not so hard that they give up. It’s a fine line.”

#### Implications for XR Design

##### Overview

The clinical interviews offered direct, practice-based insights into how digital tools can better support pediatric neurorehabilitation, particularly in relation to fatigue, motivation, and therapy adherence. “Short, modular sessions that can scale up or down are essential,” one therapist emphasized.

##### Short, Adaptive, and Engaging Sessions

Clinicians consistently highlighted the need to limit session length, especially during cognitively demanding or vision-intensive tasks. “Some days they can do 20 minutes, some days only 10,” one therapist explained, reinforcing the need for flexibility and day-to-day adaptation.

##### Detection of and Adjustment for Fatigue

As fatigue is rarely verbalized by children and may manifest subtly, the design emphasizes the value of objective movement tracking (eg, reduced limb use, slowed reactions, visual disengagement) to help infer fatigue and adapt the activity accordingly—by pausing, modifying difficulty, or prompting breaks. “If the child is zoning out, not following instructions, or slumping, those are signs.”

##### Clear, Motivating Progress Feedback

To maintain engagement, particularly in children with low frustration tolerance, progress should be immediately visible and meaningful. Gamified elements such as point tracking, stars, or personalized progress indicators were integrated to foster a sense of mastery without overreliance on external rewards. “They like seeing points. They like setting small records for themselves.”

Given the frequent balance and sensory challenges described, AR—in which children maintain visibility of their surroundings—was prioritized over full VR. This approach supports safer movement and reduces disorientation, making it more appropriate for active therapy contexts. “If you remove sight and they already have poor balance, that’s dangerous.” Although the XR prototype did not include fully customizable themes, it incorporated interaction mechanics inspired by children’s preferences—specifically, a shooter-style design based on feedback from earlier workshops. This decision aimed to align gameplay with familiar and motivating interaction styles. The system also introduced scalable difficulty through selectable target heights and adjustable game speed, allowing sessions to be tailored to the child’s physical ability and energy level. This modular structure accommodates day-to-day variability and provides therapists with flexibility in adjusting challenge levels. “Let them choose something that feels familiar and fun to them,” one therapist said, emphasizing the importance of matching activities to the child’s capacity and interests.

### Stakeholder Perceptions of Fatigue and Engagement During the Co-Design Workshops and After the XR Sessions

#### Overview

**Figure 2:**
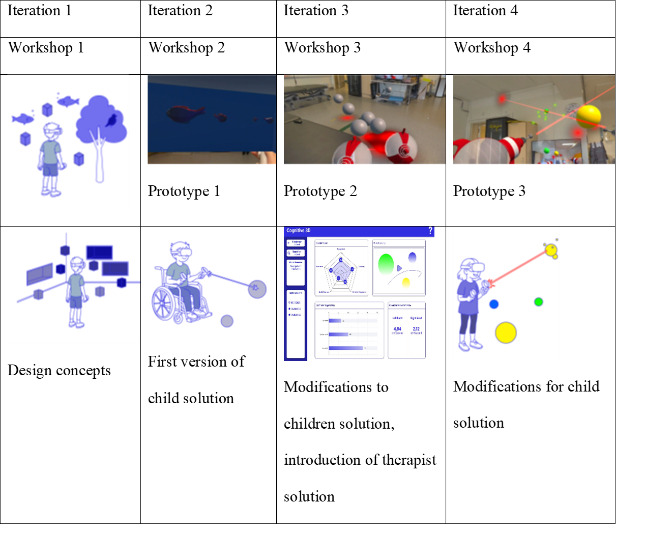
Overview of the iterative development of the extended reality therapeutic prototype for pediatric rehabilitation.

The design prototype was modified iteratively after each workshop based on feedback from participating stakeholders (see Figure 2).

#### Results of Workshop 1

The forest, fish, and cube-sorting activity concepts (Figure 2) were developed based on observations from the workshop with healthy children, particularly noting their excitement when interacting with paper prototypes created by design students. During the focus group discussion, the clinical team endorsed the need for an AR-based approach, particularly for screening symptoms such as double vision or cognitive fatigue. The forest and fish concepts were praised for their calming and accessible environments, especially suitable for nonverbal or easily fatigued children. The cube-sorting activity was appreciated for its complexity but was noted to require adjustable difficulty settings. Concerns included potential overstimulation and visual fatigue, particularly for children with preexisting vision impairments. Nonetheless, the use of Cognitive3D for postsession analytics was viewed as a promising tool for visualizing gaze and movement patterns. The clinical team emphasized the importance of short, flexible activities aligned with each child’s clinical profile.

#### Results of Workshop 2

The first prototype was designed to ensure low cognitive and physical load, enabling both children to complete the sessions without observable fatigue. However, it was perceived as overly passive. Feedback from the children and video analysis highlighted the need for more interactive gameplay. The underwater setting—particularly the wall texture—impaired spatial awareness. “I could not see my hands in the game, they were behind the blue walls,” one child noted, describing feelings of disorientation and discomfort. Engagement scores were low, with children describing the experience as repetitive. “It got boring quick, it would be nice if I could interact with the fish in some way.” Although fatigue scores were low, the experience lacked sufficient stimulation to sustain attention and engagement.

Accessibility emerged as a key concern. A child using a wheelchair required assistance to remain oriented within the environment, which reduced independence during gameplay. “The game should be able to be played by children that are in wheelchairs and children that can walk,” a therapist emphasized. The clinical team recommended shorter sessions, clearer onboarding procedures, and a simplified user interface (UI) to enhance usability. Although Cognitive3D provided valuable postsession analytics, its complexity limited feasibility for routine clinical use. Therapists highlighted the importance of appropriate pacing, personalization, and aligning gameplay with individualized therapeutic goals, such as bilateral hand use or visual tracking. Overall, the feedback underscored the need for XR systems that are inclusive, adaptable, and practical for everyday clinical contexts.

#### Implications for the Design Elements

The first prototype successfully ensured a low cognitive and physical load, which was important for safety. Both children completed the session without signs of strain or fatigue. However, the overall experience was perceived as too passive and insufficiently engaging. Feedback from the children clearly indicated a need for more interactive gameplay—something that encouraged active participation rather than passive observation. The underwater environment, particularly the wall texture, negatively affected spatial awareness. Children reported that they could not see their hands and felt visually disconnected from their surroundings. This contributed to confusion and likely reduced comfort and enjoyment. Accessibility emerged as a significant concern. One child who used a wheelchair required assistance from an adult to rotate and remain oriented during gameplay. This reliance on external assistance made the experience less fluid and diminished the sense of independence. As a result, therapists emphasized the importance of designing XR games that can be comfortably played both while seated and while standing. Interactions should be optimized for a full range of motion within a fixed orientation, reducing the need for physical repositioning or full-body turning. Therapists supported the children’s feedback and reinforced the importance of shorter, focused sessions, clearer onboarding instructions, and a simplified interface for accessing behavioral data. While the Cognitive3D tracking system offered promising analytics, its current output was considered too complex for daily therapeutic use without further simplification.

#### Results of Workshop 3

To improve engagement, the AR environment was redesigned from an underwater theme to a minimalist space that preserved visibility of the surroundings, addressing safety concerns. Workshop 3 introduced dual laser blasters, allowing children to shoot moving orbs in real time, thereby encouraging bilateral arm use and greater physical engagement. Although adaptive difficulty was not implemented, the goal was to validate more engaging mechanics. A therapist-facing dashboard was also developed to streamline monitoring, enabling profile management and session tracking.

Children reported improved usability and engagement. “I liked the game more than the fish one, this was fun.” They felt more “in the game,” appreciated the intuitive layout, and suggested adding more visual variety. “I would like more colors, the grey balls were boring.” Fatigue responses were low, and the 5-10-minute sessions were well tolerated. Therapists observed increased laughter, activity, and focus. “You could clearly see that the children were more engaged now.” They praised the dashboard’s clarity and usefulness. “This makes it much easier to understand what’s going on during the session. It’s visual, not just numbers.” However, they noted the need for clearer onboarding and session flow. “There should be some kind of menu in the game. Right now, it just keeps going.” Therapists also emphasized the importance of adaptive difficulty and personalization. “Now all the balls were low. It would be better if they could also appear near the ceiling.” Gamified elements such as scoring were recommended. “Adding points would be a good idea.” A presession menu was also suggested. “There should be a menu where they can see their points and choose what game modes they want to play.” While XR showed promise for assessments, further validation was needed. Overall, the prototype demonstrated growing clinical relevance and improved usability.

Laser-based mechanics successfully engaged both arms, offering therapeutic value for bilateral motor activity. However, despite increased interaction and interest, the lack of visual variety and feedback limited the depth of engagement over time. While children found the game fun and easier to use, they also expressed a desire for more dynamic and colorful elements to sustain their interest longer. From a clinical perspective, the prototype demonstrated improved engagement without triggering signs of fatigue. Session duration remained appropriate, and therapists observed increased movement, focus, and enthusiasm. However, their feedback underscored the need for continued development in 2 areas: adaptive difficulty to match individual abilities and more intuitive onboarding to support independent use.

#### Implications for Design Elements

This iteration addressed core issues: adding real-time target shooting and removing disorienting visuals made play more intuitive, stimulating, and physically engaging, thereby improving usability, enjoyment, and willingness to continue. Showing real-world surroundings reduced spatial confusion and overstimulation; children felt grounded and in control, and laser-based mechanics engaged both arms for bilateral practice. However, limited visual variety and feedback constrained sustained engagement; children wanted more dynamic, colorful elements. Clinically, engagement improved without fatigue, with appropriate session length and increased movement, focus, and enthusiasm. Therapists noted the need for adaptive difficulty and more intuitive onboarding to support independent use. Overall, basic gamification and a simplified UI boosted engagement without compromising comfort or safety, while highlighting the need for personalization, clearer guidance, and richer visuals in the next prototype.

#### Results of Workshop 4

The third prototype introduced several new features designed to enhance therapeutic value, interactivity, and user feedback. To boost engagement, colorful animations and a scoring system were added. A UI counter displayed scores in real time, with yellow, blue, and green balls worth 1, 2, and 3 points, respectively. Children found the game more immersive and intuitive: “I liked the game more than the fish one, this was fun.” “I want to play again to beat my score!” “I liked the colors and explosions as I shot the balls.” Therapists observed improved focus and enthusiasm. “The score made them want to play again.” Short 3-minute sessions allowed breaks. “The shorter sessions gave them a natural break.” The main menu and UI were praised: “With these UI elements this game could be played at home, not just here in therapy sessions.” Therapists also suggested customizable themes and a wider range of motion: “This would support more emotionally resonant and individualized therapy experiences.” “There should be a menu where they can see their points and choose what game modes they want to play.” Thus, children reported markedly higher enjoyment and motivation than in earlier builds, citing the visible score, colorful animations, and varied effects as making it feel like a real game rather than a clinical task. They felt in control, found the interface clearer, and especially liked tracking progress with the score, which encouraged replay (“I want to play again to beat my score!”). They rated immersion, visuals, and fun highly, with low fatigue scores; added interactivity did not cause strain, and they appeared more energized and focused. Therapists observed clear gains in engagement, usability, and therapeutic relevance, attributing improvements to the scoring mechanics, playful animations, and simplified, intuitive UI. Shorter 3-minute bouts provided natural breaks and better pacing, and the main menu plus clear UI elements supported independent use at home: “The score made them want to play again”. They recommended personalization via interchangeable themes (eg, planets, animals, fantasy elements) to match individual interests and proposed a mode that spawns targets at 3 heights simultaneously to elicit broader upper-limb movements and build flexibility, strength, and coordination.

#### Implications to the Design

The last prototype introduced several new features designed to enhance therapeutic value, interactivity, and user feedback. A key addition was the implementation of UI elements that allowed players, or therapists assisting them, to select between different game modes. Specifically, users could now choose where the balls would spawn: at floor level, mid-room, or near the ceiling. This feature was designed to introduce a scalable difficulty system by targeting different physical movement ranges. For example, children with limited shoulder mobility or upper-limb fatigue could practice raising their arms within a controlled, motivating context, supporting rehabilitation of specific motor functions. To improve engagement and visual interest, more colors and animations were added throughout the game. A scoring system was also introduced to provide players with immediate feedback and encourage repeated play. A small, nonintrusive UI counter was placed within the player’s field of view, displaying their current score in real time. The gray balls were replaced by yellow, blue, and green balls. Each yellow ball was worth 1 point, blue balls were worth 2, and green balls, being the rarest, earned 3 points. This variation added a layer of strategy and excitement to the gameplay while supporting differentiated motor effort. Each game session was set to last 3 minutes. Upon completion, the final score was displayed, and players were returned to a simple main menu interface, where they could choose to begin another round. These updates were designed to balance therapeutic intent with replayability, providing a flexible and motivating structure for both in-clinic and independent use.

### Engagement and Fatigue Across the Workshops

**Figure 3:**
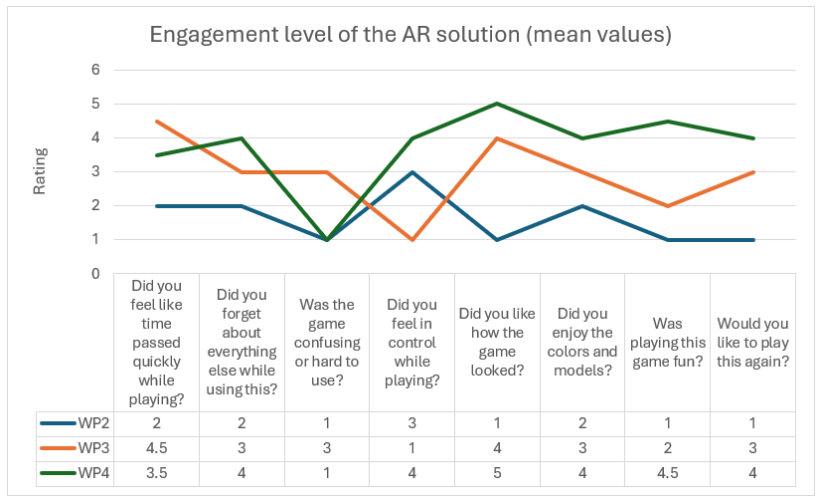
Engagement levels reported by children across the co-design workshops. WP: workshop.

**Figure 4:**
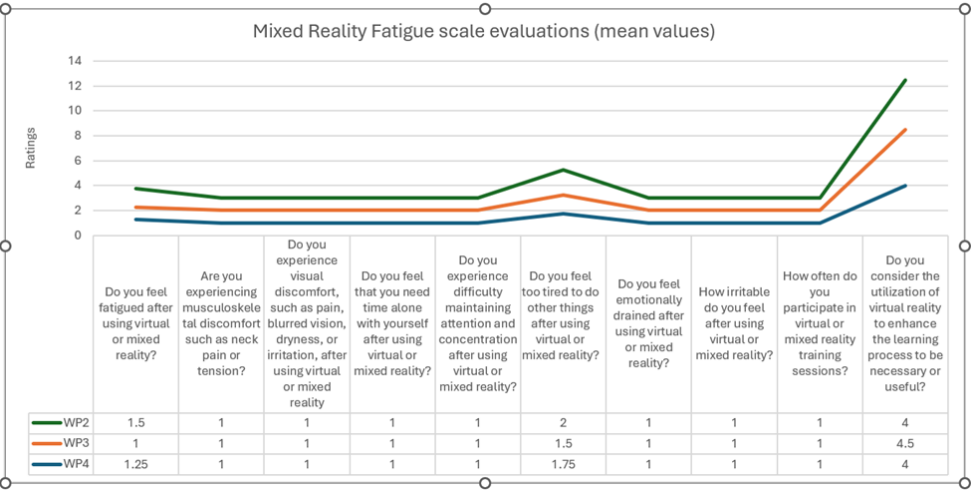
Fatigue scores reported by children across the co-design workshops.

When examined across workshops, children’s self-reported responses reveal a converging pattern of increasing engagement alongside consistently low fatigue, supporting the qualitative observations described above and illustrated in summary Figures 3 and 4. Mean engagement scores steadily increased from workshop 2 through workshop 4, reflecting improvements in perceived immersion, control, enjoyment, and motivation as iterative design changes introduced interactive mechanics, simplified visual environments, and gamified feedback. At the same time, mean fatigue-related scores remained low and showed a slight downward trend, indicating that rising engagement was not accompanied by increased physical or cognitive strain. Importantly, children’s responses to the session-length question contextualize these findings: in workshops 2 and 3, children indicated that XR sessions should not exceed 5-10 minutes, while in workshop 4, preferences shifted toward even shorter, clearly bounded sessions of approximately 3-5 minutes. This convergence suggests that short, modular exposure durations played a critical role in maintaining comfort while supporting engagement. Together, these findings demonstrate that engagement and fatigue can be decoupled through careful XR design: interactive and emotionally supportive gamification elements can enhance motivation when combined with visually grounded AR environments, simplified interfaces, adaptive pacing, and deliberately short session lengths. The alignment between children’s self-reported responses, therapist observations, and iterative design decisions underscores the importance of fatigue-sensitive session structuring as a core principle for pediatric XR-based neurorehabilitation.

## Discussion

### Principal Findings

This study demonstrates that XR-based rehabilitation, when co-designed with children and therapists, can support engagement and fatigue-aware use by enabling short sessions, visual grounding, and clinician observation of fatigue-related cues during interaction in pediatric brain injury contexts.

Iterative co-design with children, parents, and clinicians led to critical design adaptations that improved usability, personalized challenge levels, and alignment with therapeutic goals. In our study, fatigue was consistently identified as a central barrier to therapy, often expressed behaviorally rather than verbally. Fatigue emerged as the most pervasive barrier to rehabilitation, typically expressed through subtle behavioral changes rather than direct verbal reporting by children. High engagement enhanced participation but also increased the risk of overstimulation, revealing a delicate balance in which engagement must be continuously regulated to prevent fatigue-related disengagement. Therapist observations and behavioral data revealed fatigue-related changes in movement, gaze, and responsiveness that were not consistently captured through children’s self-reported fatigue ratings.

Clinicians emphasized the need for short, adaptive sessions tailored to fluctuating energy levels. Minimal gamification elements, such as scoring and visual feedback, increased motivation and replay desire when designed to support mastery rather than cognitive or sensory stimulation. Children showed noticeably higher engagement with prototypes that included interactive game mechanics (eg, target shooting) and environments that preserved visibility of their own bodies and surroundings. These features improved usability and reduced disorientation. Short, modular sessions (3-10 minutes) supported fatigue management, allowing children to complete sessions without signs of visual or physical fatigue, while therapists observed increased focus and participation. Gamification was effective when purposeful and balanced. Simple scoring systems, visual effects, and immediate feedback increased motivation without overstimulation. Customization and adaptability were also crucial; engagement improved when children could influence the experience (eg, selecting target heights). Therapists suggested further personalization to align with individual abilities and interests. Fatigue could be inferred through behavioral cues. While subjective fatigue scores were low, therapist observations and Cognitive3D tracking data revealed changes in movement, visual focus, and responsiveness as indicators of cognitive strain. Finally, AR-based designs that preserved visibility of the child’s body and surroundings reduced disorientation and supported both engagement and fatigue management, compared with immersive VR environments. Together, these findings highlight the importance of simplicity, interactivity, visual grounding, and short bursts of gameplay in XR design for pediatric neurorehabilitation.

### Comparison With Prior Work

Early case-based research using VR for pediatric ABI rehabilitation has revealed both the potential and concerns related to immersive game-based rehabilitation solutions. One of the main concerns is that, while systems such as the Xbox Kinect could improve balance performance, their cognitive, sensory, and motor demands were often poorly matched to children early in rehabilitation, leading to overstimulation, fluctuating performance, and variable motivation [36]. Our findings build directly on these observations by showing how fatigue becomes visible through behavioral changes during XR interaction and by demonstrating how simplified, visually grounded, and adaptive XR designs can preserve both engagement and safety. Our results suggest that effective XR rehabilitation for children with brain injury requires not only motivating content but also fatigue-sensitive system design that aligns with the child’s moment-to-moment capacity.

Previous research has demonstrated that fatigue is strongly associated with reduced participation and diminished quality of life in adolescents and young adults with ABI [37,38]. In particular, it has been shown that higher fatigue corresponds to greater participation restrictions, highlighting fatigue as a critical therapeutic target [38]. Building on this work, our study extends the field by examining how fatigue manifests during rehabilitation activities themselves, revealing that fatigue emerges through moment-to-moment behavioral changes rather than solely through retrospective self-report. This study advances the design of XR-based rehabilitation tools for children with ABIs by foregrounding fatigue as a central design constraint—an area often underemphasized in prior work.

Consistent with Rohrer-Baumgartner et al [3] and Mazzone et al [21], our findings confirm that fatigue in pediatric brain injury is multifaceted—cognitive, physical, emotional, and sensory—and often behaviorally expressed rather than verbally reported. Our results show that XR-based interaction enables real-time observation of fatigue-related behaviors during task engagement, offering a concrete way to surface these otherwise hidden indicators. In line with Mazzone et al [21], whose findings indicate that clinicians depend heavily on behavioral cues rather than child self-report to detect fatigue, our results suggest that XR-based behavioral markers, such as gaze, posture, and response latency, can support this observational process in a structured manner.

In line with Mazzone et al [21], whose findings indicate that clinicians often rely on behavioral cues rather than child self-report to identify fatigue following pediatric brain injury, this study demonstrates how XR-based rehabilitation can support this observational process in a structured yet clinically aligned manner. By capturing changes in gaze, posture, movement, and responsiveness during short, adaptive XR sessions, the system allowed fatigue to become visible without requiring children to articulate their internal state. Importantly, these behavioral indicators did not replace clinical judgment but supported shared understanding and collaborative decision-making among therapists, children, and families. Together, these findings position XR not as a fatigue-reducing technology per se, but as a fatigue-sensitive design approach that operationalizes existing clinical practices while preserving engagement and emotional safety. Clinicians also need to continually adjust rehabilitation demands to manage fatigue [21]. Our findings extend this work by illustrating how adaptive XR session length and task difficulty can embed this clinical reasoning directly into the intervention design.

While Wender et al [20] emphasize the need for adaptive systems to prevent disengagement, few studies operationalize this need through concrete design strategies. Our work builds on this by implementing modular, short-duration sessions (3-10 minutes) and adaptive difficulty scaling, which allowed children to complete tasks without signs of visual or cognitive overload. While Proschowsky et al [37] describe gradual changes in fatigue across the rehabilitation period using standardized measures, our results suggest that clinically relevant fatigue fluctuations occur within sessions and are primarily observable through changes in interaction behavior, underscoring the need for complementary real-time monitoring approaches. Our approach also aligns with Sweller’s [35] Cognitive Load Theory, which advocates minimizing extraneous load to preserve mental resources—particularly critical in pediatric neurorehabilitation.

Proschowsky et al [37] also demonstrated a consistent discrepancy between child and parent fatigue ratings, particularly for cognitive fatigue, suggesting that children may lack insight into their own fatigue experience. Our findings extend this work by showing that XR-based behavioral indicators—such as gaze disengagement, postural changes, and response latency—can provide an additional, nonverbal layer of information that aligns with clinicians’ observational fatigue assessments. In contrast to XR systems with fixed difficulty levels (eg, [11]), our prototype dynamically adjusted the challenge based on fatigue cues, supporting the “just-right challenge” principle from occupational therapy [39]. This approach not only sustained engagement but also improved therapeutic focus, as observed by clinicians. The use of AR over VR—supported by Rebenitsch and Owen [40]—further reduced disorientation and preserved environmental awareness, which is especially important for children with balance or visual sensitivities. Gamification was another key differentiator. While Deterding et al [41] and O’Brien et al [32] highlight the motivational benefits of game elements, our study extends this by emphasizing adaptive gamification. Rather than static rewards or competitive mechanics [42], we prioritized emotionally supportive feedback, autonomy, and visual grounding. Children responded positively to features such as target selection, score feedback, and interactive animations, confirming that gamification enhances motivation when developmentally appropriate and cognitively balanced [43].

### Limitations and Future Work

As a qualitative case study, this research is context-specific and not intended for statistical generalization [27,29]. The small participant sample, drawn from a single clinical setting, limits transferability. While including healthy children in early prototyping aligns with child-centered design practices [44,45], it may have introduced discrepancies between early feedback and the needs of children with ABIs. This study used Design Thinking [46] within a qualitative case study to guide XR development for children with brain injuries. Its 5 phases—empathize, define, ideate, prototype [47,48], and test—structured co-design workshops and ensured stakeholder insights shaped solutions focused on fatigue detection, adaptive engagement, and emotional safety. In the empathize phase [49], interviews, observations, and shadowing sessions with clinicians, parents, and children were conducted to understand clinical routines, fatigue triggers, and children’s emotional responses to therapy. In the define phase [47], these insights were synthesized into problem statements, such as the need for understanding fatigue and safeguarding mechanisms to prevent overstimulation. During the ideate phase [50], multidisciplinary workshops with clinicians, designers, and children generated concepts ranging from environmental cues for fatigue to customizable levels of XR immersion. In the prototyping phase [47], low- and mid-fidelity XR mockups were created and iteratively refined based on stakeholder feedback, focusing on interaction patterns, visual clarity, and observational insights. Finally, in the test phase [51], preliminary XR prototypes were trialed in simulated therapy sessions, where children and clinicians evaluated usability, comfort, and safety, directly informing adjustments such as simplified interfaces and adaptive pacing.

This approach was seen as particularly valuable in pediatric rehabilitation, where the wide variation in children’s physical, emotional, and cognitive abilities calls for personalized and context-sensitive design [52]. Co-design is a participatory design approach that involves end users and stakeholders as active contributors in the design process, rather than passive recipients of a finished product. According to Sanders and Stappers [53], co-design centers on collaboration, mutual learning, and shared ownership, enabling users to bring their experiences, needs, and creativity into the creation of meaningful solutions. In our study, Design Thinking was used as an organizing framework to structure the co-design and iterative prototyping process, ensuring that stakeholder insights were translated into concrete, testable design changes [54]. Across empathize, define, ideate, prototype, and test, the focus was to reduce participant burden, capture fatigue-aware requirements, and iteratively refine engagement-related mechanics in short XR sessions. The choice of a case study combined with co-design is justified by the exploratory nature of the research. Together, design thinking, co-design, and case study provide a robust methodology for integrating multiple stakeholder perspectives while iteratively shaping and evaluating the prototype within its intended context of use. From a process perspective, the Design Thinking structure strengthened traceability between stakeholder needs and design outcomes [55]: the empathize and define phases foregrounded fatigue-aware constraints (session duration, sensory load, safety), ideation clarified interaction preferences, and prototype/test cycles enabled rapid validation of engagement mechanics (eg, interactive shooting and scoring) while maintaining visual grounding through AR. This helped balance engagement with the risk of overstimulation, which clinicians described as a central tension in pediatric ABI rehabilitation. Design Thinking supported rapid iteration and stakeholder alignment; however, the iterative nature also meant that not all clinician suggestions could be implemented and retested within the study time frame. Future work should evaluate the refined design elements across more sites and over longer home-use periods. Feedback from the clinical team, though rich, was collected after the final prototype iteration and therefore could not be integrated, tested, or clinically validated. Future work should also explore personalization options (eg, themes, interaction styles) and multilevel interaction design to support both fine and gross motor skills. Prior research emphasizes the importance of individualized therapy content for sustaining motivation and improving outcomes [11,26]. Although the initial prototype dashboard was intended to visualize behavioral fatigue indicators over the long term (eg, gaze, posture), this was not implemented in the present design phase, and the system also lacked physiological data integration. Future systems could integrate XR-compatible wearables to capture biometric signals, such as heart rate variability or pupil dilation, enhancing fatigue detection [56,57]. The dashboard could be further developed as a fatigue detection tool or as a clinical decision-support tool with customizable visualizations, engagement tracking, and real-time alerts [25]. While this study focused on behavioral indicators of fatigue observable during XR interaction, future research may explore the potential role of additional physiological signals, such as heart rate variability or pupil dilation, as complementary sources of information.

Although fatigue detection was not conducted in this study, it is important to note that evidence supporting the validity and reliability of these biometric measures for fatigue detection in pediatric ABI populations is currently limited [37]. Our study revealed a need for objective fatigue detection methods that could support safe personalization of XR solutions. Existing research on biometric fatigue markers, however, stems largely from adult neurological populations or from studies of cognitive load and fatigue in other clinical or nonclinical contexts [58]. As such, any integration of physiological signals into pediatric XR rehabilitation systems should be considered exploratory and would require careful validation, ethical consideration, and age-appropriate interpretation before clinical use. To improve generalizability, future studies should include more diverse participants, test across multiple sites, and assess long-term therapeutic impact. Integration with clinical data systems may also be necessary for sustainable implementation in routine care.

## Data Availability

The raw data cannot be made publicly available due to the involvement of a vulnerable population. It is important that the data remain unpublished to prevent potential identification of the children involved in the study.

## Appendix

Multimedia Appendix 1 Survey questions.

Multimedia Appendix 2 Workshop and interview guide.

Multimedia Appendix 3 Data analysis themes.

Multimedia Appendix 4 Participant flow diagram.

## Abbreviations

ABI: acquired brain injury
AR: augmented reality
GDPR: General Data Protection Regulation
SIKT: Norwegian Agency for Shared Services in Education and Research
UES: User Engagement Scale
UI: user interface
VMRFS: Virtual and Mixed Reality Fatigue Scale
VR: virtual reality
XR: extended reality

## References

[1] Anderson V, Le Brocque R, Iselin G, Eren S, Dob R, Davern TJ, McKinlay L, Kenardy J (2012). Adaptive ability, behavior and quality of life pre and posttraumatic brain injury in childhood. Disabil Rehabil.

[2] Catroppa C, Anderson V (2004). Recovery and predictors of language skills two years following pediatric traumatic brain injury. Brain Lang.

[3] Rohrer-Baumgartner N, Laberg Holthe I, Svendsen EJ, Dahl HM, Borgen IMH, Hauger SL, Thulesius MS, Wade SL, Røe Cecilie, Løvstad Marianne (2025). Children and families with chronic pediatric acquired brain injury in need of rehabilitation: characteristics and main challenges in daily life. Disabil Rehabil.

[4] Holthe IL, Dahl HM, Rohrer-Baumgartner N, Eichler S, Elseth MF, Berntsen T, Yeates KO, Andelic N, Løvstad Marianne, Holthe (2021). Neuropsychological impairment, brain injury symptoms, and health-related quality of life after pediatric TBI in Oslo. Front Neurol.

[5] Beretta E, Cesareo A, Biffi E, Schafer C, Galbiati S, Strazzer S (2018). Rehabilitation of upper limb in children with acquired brain injury: a preliminary comparative study. J Healthc Eng.

[6] Yenilmez O, Altug F (2026). Effect of adding virtual reality to individualized exercise therapy on gross motor function, balance, and functional mobility in children with hemiparetic cerebral palsy: a randomized single-blinded controlled trial. Clin Pediatr (Phila).

[7] Tatla SK, Sauve K, Jarus T, Virji-Babul N, Holsti L (2014). The effects of motivating interventions on rehabilitation outcomes in children and youth with acquired brain injuries: a systematic review. Brain Inj.

[8] Janssen J (2017). Gamification in physical therapy: more than using games. Pediatric Physical Therapy.

[9] Choi JY, Yi S, Shim D, Yoo B, Park ES, Rha D (2023). Home-based virtual reality-enhanced upper limb training system in children with brain injury: a randomized controlled trial. Front Pediatr.

[10] Malick WH, Butt R, Awan WA, Ashfaq M, Mahmood Q (2022). Effects of augmented reality interventions on the function of upper extremity and balance in children with spastic hemiplegic cerebral palsy: a randomized clinical trial. Front Neurol.

[11] Laver K.E., Lange B., George S., Deutsch J. E., Saposnik G., Chapman Madison, Crotty Maria (2025). Virtual reality for stroke rehabilitation. Cochrane Database Syst Rev.

[12] Power M, Kennedy S, Cleary F, Mills I, Kinsella S, Celdrán AH (2024). A systematic literature review of XR interventions to improve motor skills development among autistic children. IEEE Access.

[13] Keshner EA, Kenyon RV (2004). Using immersive technology for postural research and rehabilitation. Assist Technol.

[14] Ren Z, Wu J (2019). The effect of virtual reality games on the gross motor skills of children with cerebral palsy: a meta-analysis of randomized controlled trials. Int J Environ Res Public Health.

[15] Wu J, Loprinzi PD, Ren Z (2019). The rehabilitative effects of virtual reality games on balance performance among children with cerebral palsy: a meta-analysis of randomized controlled trials. Int J Environ Res Public Health.

[16] Ponsford J, Schönberger Michael, Rajaratnam Shantha M W (2015). A model of fatigue following traumatic brain injury. J Head Trauma Rehabil.

[17] Shahid A, Shen J, Shapiro CM (2010). Measurements of sleepiness and fatigue. J Psychosom Res.

[18] Gawron VJ (2017). Overview of self-reported measures of fatigue. The International Journal of Aviation Psychology.

[19] Riccardi J, Ciccia Angela (2021). Cognitive fatigue in pediatric traumatic brain injury: a meta-analysis and scoping review. J Head Trauma Rehabil.

[20] Wender CLA, Farrar E, Sandroff BM (2025). Attrition, adherence, and compliance to exercise training interventions in persons with traumatic brain injury: a systematic review of training studies. Brain Inj.

[21] Mazzone O, Conroy R, Jenkin T, Scheinberg A, Knight S (2025). The assessment and management of fatigue following paediatric acquired brain injury: rehabilitation clinicians' perspectives. Neuropsychol Rehabil.

[22] Khan F, Amatya B (2018). Management of fatigue in neurological disorders: implications for rehabilitation. Journal of the International Society of Physical and Rehabilitation Medicine.

[23] Danson E, Brunsdon R, Lane-Brown A, Oxenham V (2026). Neuropsychol Rehabil.

[24] Montoya MF, Munoz JE, Henao OA (2020). Enhancing virtual rehabilitation in upper limbs with biocybernetic adaptation: the effects of virtual reality on perceived muscle fatigue, game performance and user experience. IEEE Trans Neural Syst Rehabil Eng.

[25] Annabestani M, Sriram S, Caprio A, Janghorbani S, Wong SC, Sigaras A, Mosadegh B (2024). High-fidelity pose estimation for real-time extended reality (XR) visualization for cardiac catheterization. Sci Rep.

[26] Latulippe K, Hamel C, Giroux D (2020). Co-design to support the development of inclusive eHealth tools for caregivers of functionally dependent older persons: social justice design. J Med Internet Res.

[27] Yin R. K. (2009). Case Study Research: Design and Methods (4th edition), Vol. 5.

[28] Branca Vergano L, Monesi M, Vicenti G, Bizzoca D, Solarino G, Moretti B Journal Article Reporting Standards (JARS). American Psychological Association (APA).

[29] Lincoln YS (1985). Naturalistic Inquiry (Vol. 75).

[30] Younas A, Masih Y, Sundus A (2025). Alternatives to 'saturation' for greater transparency in reporting of sample size decision-making in qualitative research. Evid Based Nurs.

[31] Creswell JW, Miller DL (2000). Determining validity in qualitative inquiry. Theory Into Practice.

[32] O’Brien HL, Cairns P, Hall M (2018). A practical approach to measuring user engagement with the refined User Engagement Scale (UES) and new UES short form. International Journal of Human-Computer Studies.

[33] Cintora-Sanz A, Muñoz-Romo Raúl, Schrom-Feiertag Helmut, Blanco-Lara Alberto, Vazquéz-Rodriguez Tatiana, Cardós-Alonso M Carmen (2026). BMC Med Educ.

[34] Braun V, Clarke V (2008). Using thematic analysis in psychology. Qualitative Research in Psychology.

[35] Sweller J (2010). Cognitive load during problem solving: effects on learning. Cognitive Science.

[36] Cheung J, Maron M, Tatla S, Jarus T (2013). Virtual reality as balance rehabilitation for children with brain injury: a case study. TAD.

[37] Proschowsky MS, Reimers SH, Granhøj Anette (2024). Fatigue among children and adolescents with acquired brain injury in a specialized neurorehabilitation setting. Front Rehabil Sci.

[38] van Markus-Doornbosch F, van der Holst M, de Kloet AJ, Vliet Vlieland TPM, Meesters JJL (2020). Fatigue, participation and quality of life in adolescents and young adults with acquired brain injury in an outpatient rehabilitation cohort. Dev Neurorehabil.

[39] Christie A (2002). A meaningful occupation: the just right challenge. Aus Occup Therapy J.

[40] Rebenitsch L, Owen C (2016). Review on cybersickness in applications and visual displays. Virtual Reality.

[41] Deterding S (2011). From game design elements to gamefulness: defining "gamification".

[42] Hallifax S (2019). Adaptive gamification in education: a literature review of current trends and developments.

[43] Al-Khresheh MH (2025). The cognitive and motivational benefits of gamification in English language learning: a systematic review. TOPSYJ.

[44] Scaife M, Rogers Y (1998). Kids as informants: telling us what we didn't know or confirming what we knew already?. The Design of Children's Technology.

[45] Druin A (2002). The role of children in the design of new technology. Behaviour & Information Technology.

[46] Brown T (2008). Design thinking. Harv Bus Rev.

[47] Rösch N, Tiberius V, Kraus S (2023). Design thinking for innovation: context factors, process, and outcomes. EJIM.

[48] Ku B, Lupton E (2022). Health Design Thinking: Creating Products and Services for Better Health.

[49] Li X, Chen J, Fu H (2024). The roles of empathy and motivation in creativity in design thinking. Int J Technol Des Educ.

[50] Asano Y (2023). Defining the problem’s solution to lead to the ideation phase: - a case study on the use of “how might we...”.

[51] Jaskyte K, Liedtka J (2022). Design thinking for innovation: practices and intermediate outcomes. Nonprofit Mgmnt & Ldrshp.

[52] Levac DE (2024). Individual and contextual factors influencing children's effort in pediatric rehabilitation interventions. Dev Med Child Neurol.

[53] Sanders EB, Stappers PJ (2008). Co-creation and the new landscapes of design. CoDesign.

[54] Saidi T, Mutswangwa CT, Douglas TS (2019). Design thinking as a complement to human factors engineering for enhancing medical device usability. Engineering Studies.

[55] Stefoska-Needham A, Nealon J, Charlton K, Fildes K, Lambert K (2025). Applying design thinking for co-designed health solutions: a case study on chronic kidney disease in regional Australia. Int J Environ Res Public Health.

[56] Srinivasan AG, Smith SS, Pattinson CL, Mann D, Sullivan K, Salmon P, Soleimanloo SS (2024). Heart rate variability as an indicator of fatigue: a structural equation model approach. Transportation Research Part F: Traffic Psychology and Behaviour.

[57] Martins R, Carvalho J (2015). Eye blinking as an indicator of fatigue and mental load: a systematic review. https://www.researchgate.net/publication/277010972_Eye_blinking_as_an_indicator_of_fatigue_and_mental_load_-_a_systematic_review.

[58] Adão Martins N. R., Annaheim S., Spengler C. M., Rossi R. M. (2021). Fatigue monitoring through wearables: a state-of-the-art review. Frontiers in Physiology.

