# Supplementary material for: Opportunities and Concerns of Gamified, Extended Reality for Home-Based Motor Rehabilitation for Children With Brain Injury: Qualitative Case Study on Design Elements Related to the Engagement and Fatigue Perspectives
Source: J Med Internet Res. 2026 Jun 2;28:e84013. doi: 10.2196/84013 (PMC13273198; doi:10.2196/84013)
Supplement: Multimedia Appendix 1 [file jmir_v28i1e84013_app1.docx]

| Engagement scale |  |
| --- | --- |
| Did you feel like time passed quickly while playing? |  |
| Did you forget about everything else while using this? |  |
| Was the game confusing or hard to use? |  |
| Did you feel in control while playing? |  |
| Did you like how the game looked? |  |
| Did you enjoy the colors and models? |  |
| Was playing this game fun? |  |
| Would you like to play this again? |  |

| **Mixed Reality Fatigue Scale** |  |
| --- | --- |
| Do you feel fatigued after using virtual and/or mixed reality? |  |
| Are you experiencing musculoskeletal discomfort such as neck pain or tension? |  |
| Do you experience visual discomfort, such as pain, blurred vision, dryness, or irritation, after using virtual and/or mixed reality? |  |
| Do you feel that you need time alone with yourself after using virtual and/or mixed reality? |  |
| Do you experience difficulty maintaining attention and concentration after using virtual and/or mixed reality? |  |
| Do you feel too tired to do other things after using virtual and/or mixed reality? |  |
| Do you feel emotionally drained after using virtual and/or mixed reality? |  |
| How irritable do you feel after using virtual and/or mixed reality? |  |
| How often do you participate in virtual or mixed reality training sessions? |  |
| In your opinion, a mixed reality training session (with glasses) should not exceed…? |  |
| Do you consider the utilization of virtual reality to enhance the learning process to be necessary and/or useful? |  |
