# Supplementary material for: Opportunities and Concerns of Gamified, Extended Reality for Home-Based Motor Rehabilitation for Children With Brain Injury: Qualitative Case Study on Design Elements Related to the Engagement and Fatigue Perspectives
Source: J Med Internet Res. 2026 Jun 2;28:e84013. doi: 10.2196/84013 (PMC13273198; doi:10.2196/84013)
Supplement: Multimedia Appendix 2 [file jmir_v28i1e84013_app2.docx]

WORKSHOP AND INTERVIEW GUIDE (Data Collection Form)

Background Questions for Patient:
Participant Name and Code: ____________________________________________
Gender: ____________________________________________________________
Age: _______________________________________________________________
Household Members: _________________________________________________
Child's Age: ________________________________________________________
Type of Brain Injury: ____________________________________________________
Where Did Rehabilitation Take Place: ___________________________________
Where Is Late-Stage Rehabilitation Taking Place: ___________________________

Background Questions for Healthcare Professional:
Participant Name and Code: ____________________________________________
Occupation: _________________________________________________________
Work Experience with Patients and Parents: _______________________________

Note: Not all questions are asked in every workshop or interview. The goal is to select relevant questions from the guide for each session (max 90 min).

WORKSHOP 1:

QUESTIONS FOR THERAPIST:
• Can you tell us a bit about your work with patients?
• What is important to you regarding their improvement?
• What helps them adopt new routines and stay attentive to training?
• How do you teach them these new routines?
• Do you prescribe home exercises?
• What do you see as the advantages and disadvantages of VR games for home rehabilitation?
• How useful, effective, or targeted are these exercises in your opinion?
• How safe are they for home use? How easy are they to use? How usable are they in your view?
• How should we recommend using this device (preparation, recommendations, time usage)? What would be a therapist’s role in adapting to VR training?
• Do you see any psychological/cultural or other barriers that might make AR/VR unacceptable for patients?
• How can we improve patients’ mastery experiences and motivation in VR (short and long term)?
• What do you think about patient engagement when a therapist is present versus absent?

QUESTIONS FOR PATIENTS AND RELATIVES:
• Can you tell us about your experiences, needs, and challenges in rehabilitation after your child returned home from the hospital?
• Can you describe your daily life at home?
• What kind of rehabilitation have you had since your child came home?
• What happened next?
• Can you describe how rehabilitation has been at home?
 o What kind of rehabilitation have you received?
 o What goals have you had for home rehabilitation? What has been most important for you personally?
 o Have you had any positive experiences?
 o Any challenges or difficulties where you needed more help?
• Can you describe your current situation at home?
 o What kind of rehabilitation is your child receiving now?

• What has been the most important and most difficult in everyday life and rehabilitation?
• What has been most important and most difficult for you and your child in rehabilitation?
• What is most important and most difficult for you and your child now?
• What has been most important for you and your child in home rehabilitation?
• What is difficult for you and your child in daily life? How?

• Can you tell us about your use of digital solutions or games?
• What kind of digital technology (mobile phone, PC, iPad, VR, AR) does your child use daily?
 o How do you use it? What features are useful?
• Is there a difference in how you use technology now compared to before?
• Have you used technology in your child’s home rehabilitation?
• How have you used technology in home rehabilitation?
• Have you ever played any games? Which ones? Alone, with others, or both?
• What do you think about using digital solutions, VR or AR glasses for your child’s rehabilitation?
• What kind of digital solution would you consider using for rehabilitation? What features should it have to best support your rehabilitation? Why?

We will show you these personal technologies:
• Can you tell us about your experience testing the digital solutions?
• What was your first impression? What would you use, what not, and why?
• What did you think about talking to the virtual avatar? How was it to perform the exercise?
• How would you describe communication with the avatar?
• What was good/challenging about these design solutions? How did you experience movement exercises?
• How did it feel to work on everyday tasks in real life compared to AR games? How would you describe the difference between real and VR exercises?
• What do you think about accessories like sensor gloves, smartwatches, or motion bands? (We will show pictures and explain how they work)
• What are the advantages/disadvantages of the digital solutions?
• What do you think about having an AR or video therapy group? Which would you choose and why?

How would it feel to use the solution at home?
• Have you used rehabilitation technology at home? How would it feel to use it at home? (How did it feel to prepare/handle the equipment?)
• What is useful/challenging about these solutions when you or your child use them at home?
• How can these technologies be improved? What would you do if you were the designer (draw/create it)?
• What do you think about using these technologies with family, friends, other patients, therapists?
• What do you think about meeting a therapist online or in a VR/AR world?
• How should we change these solutions to make them more suitable/acceptable/useful for you?

WORKSHOP 2

QUESTIONS FOR THERAPIST:
Can you tell us about your experiences, needs, and challenges in rehabilitation after your child returned home from the hospital?
• What is the current practice for children discharged from hospital to home?
• How are goals set and adjusted for children undergoing home rehabilitation after an accident?
• How does the interdisciplinary team communicate with the patient about rehabilitation goals?
• What takes the most time/effort/resources in your daily routine for children in home rehabilitation?
• What tasks are most challenging for children in home rehabilitation?
• Do you use personal technology with the children? What do you use most often?
• What kind of technology do you miss (that you’ve heard of) that could help you do your job better/easier/more effectively?

Can you tell us about your current use of digital solutions or games for rehabilitation of patients after trauma?
• Have you used any digital solutions with the child in hospital or at home?
• How was it to prepare the equipment?
• What was the experience with the equipment for you and the patient?
• What kind of digital solution do you think could work for home rehabilitation of trauma patients? What functionality should it have? Why?

We will show you these personal technologies:
Can you tell us about your experience testing the digital solutions?
• Could any of these be useful for you in your current or future practice?
• What advantages/disadvantages do you personally see?
• What do you think about using individual vs. group therapy for children in home rehabilitation?
• What about video therapy vs. VR therapy? How do you feel about communicating with patients through avatars?
• What situations and content are suitable for this technology?
• Are there features you find good or problematic, especially when patients are alone at home?
• What do you think about accessories like sensor gloves, smartwatches, or motion bands? Home rehabilitation situations: What do you think would happen if we provide this equipment for home use?
• What needs to change for patients to be able to use the equipment at home?
• What exercises/support for trauma patients (that we haven’t considered) could be addressed with these technologies in your opinion?
• What is useful/challenging with these tools? How can these technologies be improved?
• What do you think about patients using these technologies with family, friends, other patients, therapists?
• What do you think about meeting patients online in video therapy or in the VR world?
• What features should the solution have to support physical movements of patients at different stages of rehabilitation?
• What features could the solution have to support the patient journey?
• What features could the solution have to support the mental state of patients, such as peer support and support from therapists?

WORKSHOP 3–4

Showing technology to patients and health experts/therapists:
Can you tell us about your experience testing the digital solutions?
• How was your experience trying these solutions?
• How easy was it to use in your impression? Was the solution engaging/inviting/interesting?
• Did the solution do what you expected it to do?
• Were there any features you liked more than others or would like to use more? Any features you didn’t like or didn’t use much?
• Were there any instances where the solution seemed useful/not useful? Which ones? Was anything surprising? If yes, what?
• Was there anything you expected the solution to have that wasn’t there? Anything that didn’t look as expected? What was missing?
• What was difficult or strange about this usage scenario, if anything? What was easier?
• What was unnecessary, if anything? Was anything misplaced? What was it?
• How would you rate the difficulty of using this equipment?
• How does using VR/AR affect your experience?
• How would you rate the difficulty (scale from 0–10) for this task?

What changes do you think have occurred due to using the solution? What have you learned, what would you change?
• If you had a magic wand, what would you change about this technology?
• What changes do you think could happen due to using the solution?
• Have you learned anything after using the solution during treatment? What? Were there any specific features that were especially useful? Which ones?
• Were there any instances where the solution was not useful or showed progress?
• What features could the solution have to support physical movements of patients at different levels?
• What features could the solution have to support patient navigation?
• What features could the solution have to support the mental state of patients, such as peer support and support from therapists?

INTERVIEW WITH PATIENTS

Can you tell us about your progress and what you think about the task we tested?
• What stood out to you during the session?
• Now that some time has passed, what do you think about the task we tested?
• How would you describe your progress in relation to your goals?
• Are there any changes in progress/treatment you should consider?
• What should we (researchers) remember for next time?

Can you tell us about your experience of the usefulness of the solution?
• To what extent do you think using this solution will improve your performance related to your treatment goals, why/why not?
• To what extent do you think this solution increases effectiveness related to your treatment goals, why/why not?
• To what extent do you think using this solution will help you carry out your daily tasks, why/why not?
• To what extent do you think the solution is useful for achieving your treatment goals, why/why not?

Can you tell us about your feelings regarding the solution’s usability?
• How easy is it to use this solution, why/why not?
• How easy do you think it is to learn and use this technology?
• Would it be easy to get this solution to do what you want it to do? Why/why not?
• Would it be easy for you to become proficient in using this solution, why/why not?

Can you tell us about your intention to use the solution regularly for home rehabilitation?
• Why/why not? What should we change to make you want to use it?

What did you think about the workshops?
• Were the workshops useful for you, or would you have preferred fewer or more? Why?
