# Supplementary material for: Opportunities and Concerns of Gamified, Extended Reality for Home-Based Motor Rehabilitation for Children With Brain Injury: Qualitative Case Study on Design Elements Related to the Engagement and Fatigue Perspectives
Source: J Med Internet Res. 2026 Jun 2;28:e84013. doi: 10.2196/84013 (PMC13273198; doi:10.2196/84013)
Supplement: Supplementary file 3 [file jmir_v28i1e84013_app3.docx]

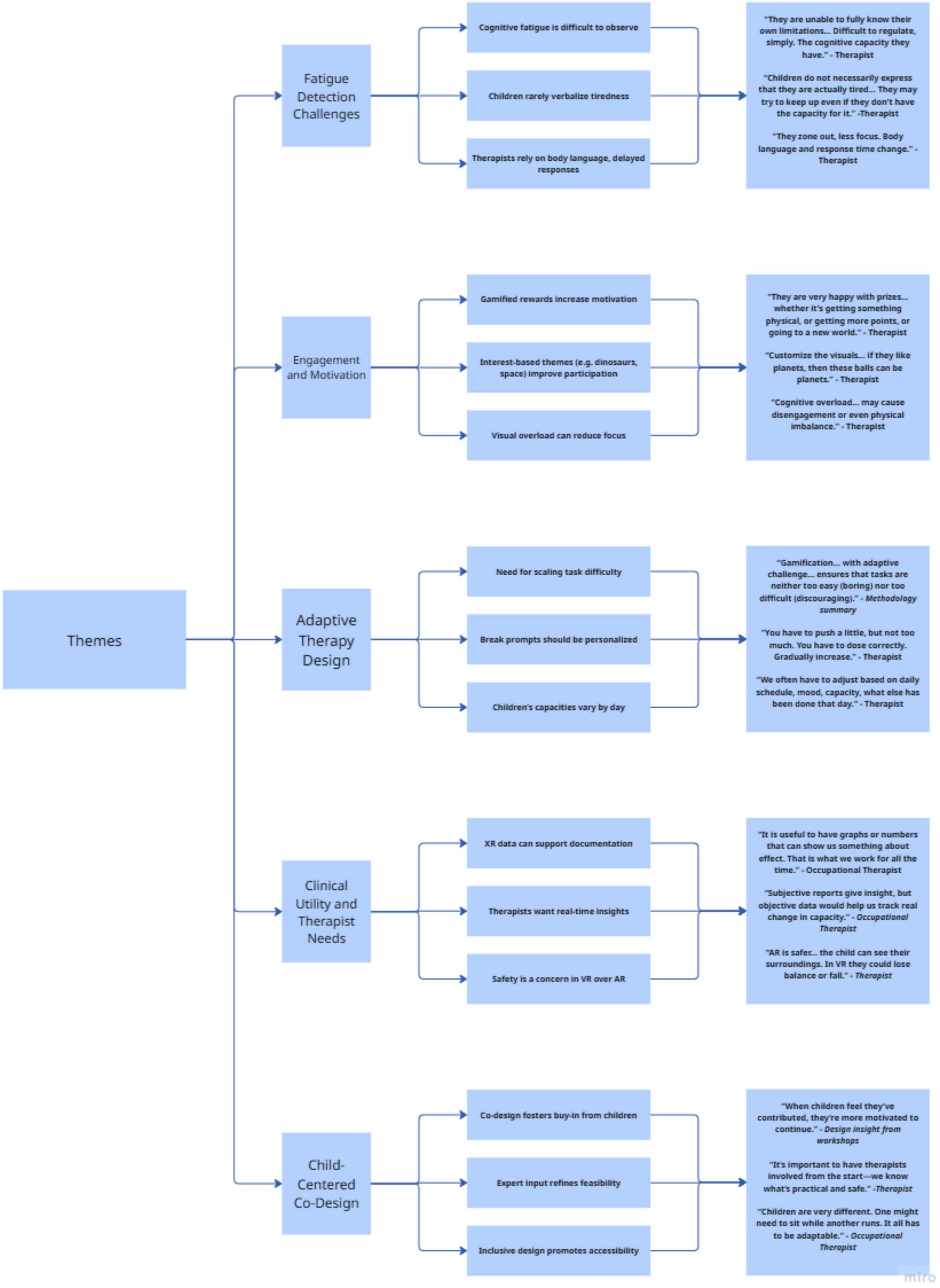

Themes
Fatigue Detection Challenges
Cognitive fatigue is difficult to observe
Children rarely verbalize tiredness
Therapists rely on body language, delayed responses
"They are unable to fully know their own limitations... Difficult to regulate, simply. The cognitive capacity they have." - Therapist
"Children do not necessarily express that they are actually tired.... They may try to keep up even if they don't have the capacity for it." -Therapist
"They zone out, less focus. Body language and response time change." - Therapist
Engagement and Motivation
Gamified rewards increase motivation
Interest-based themes (e.g. dinosaurs, space) improve participation
Visual overload can reduce focus
"They are very happy with prizes... whether it's getting something physical, or getting more points, or going to a new world." - Therapist
"Customize the visuals... if they like planets, then these balls can be planets." - Therapist
"Cognitive overload... may cause disengagement or even physical imbalance." - Therapist
Adaptive Therapy Design
Need for scaling task difficulty
Break prompts should be personalized
Children's capacities vary by day
"Gamification.... with adaptive challenge... ensures that tasks are neither too easy (boring) nor too difficult (discouraging)." - *Methodology summary*
"You have to push a little, but not too much. You have to dose correctly. Gradually increase." - Therapist
"We often have to adjust based on daily schedule, mood, capacity, what else has been done that day." - Therapist
Clinical Utility and Therapist Needs
XR data can support documentation
Therapists want real-time insights
Safety is a concern in VR over AR
"It is useful to have graphs or numbers that can show us something about effect. That is what we work for all the time." - Occupational Therapist
"Subjective reports give insight, but objective data would help us track real change in capacity." - *Occupational Therapist*
"AR is safer... the child can see their surroundings. In VR they could lose balance or fall." - *Therapist*
Child-Centered Co-Design
Co-design fosters buy-in from children
Expert input refines feasibility
Inclusive design promotes accessibility
"When children feel they've contributed, they're more motivated to continue." - *Design insight from workshops*
"It's important to have therapists involved from the start—we know what's practical and safe." -*Therapist*
"Children are very different. One might need to sit while another runs. It all has to be adaptable." - *Occupational Therapist*
miro
