# Supplementary material for: Opportunities and Concerns of Gamified, Extended Reality for Home-Based Motor Rehabilitation for Children With Brain Injury: Qualitative Case Study on Design Elements Related to the Engagement and Fatigue Perspectives
Source: J Med Internet Res. 2026 Jun 2;28:e84013. doi: 10.2196/84013 (PMC13273198; doi:10.2196/84013)
Supplement: Supplementary file 4 [file jmir_v28i1e84013_app4.docx]

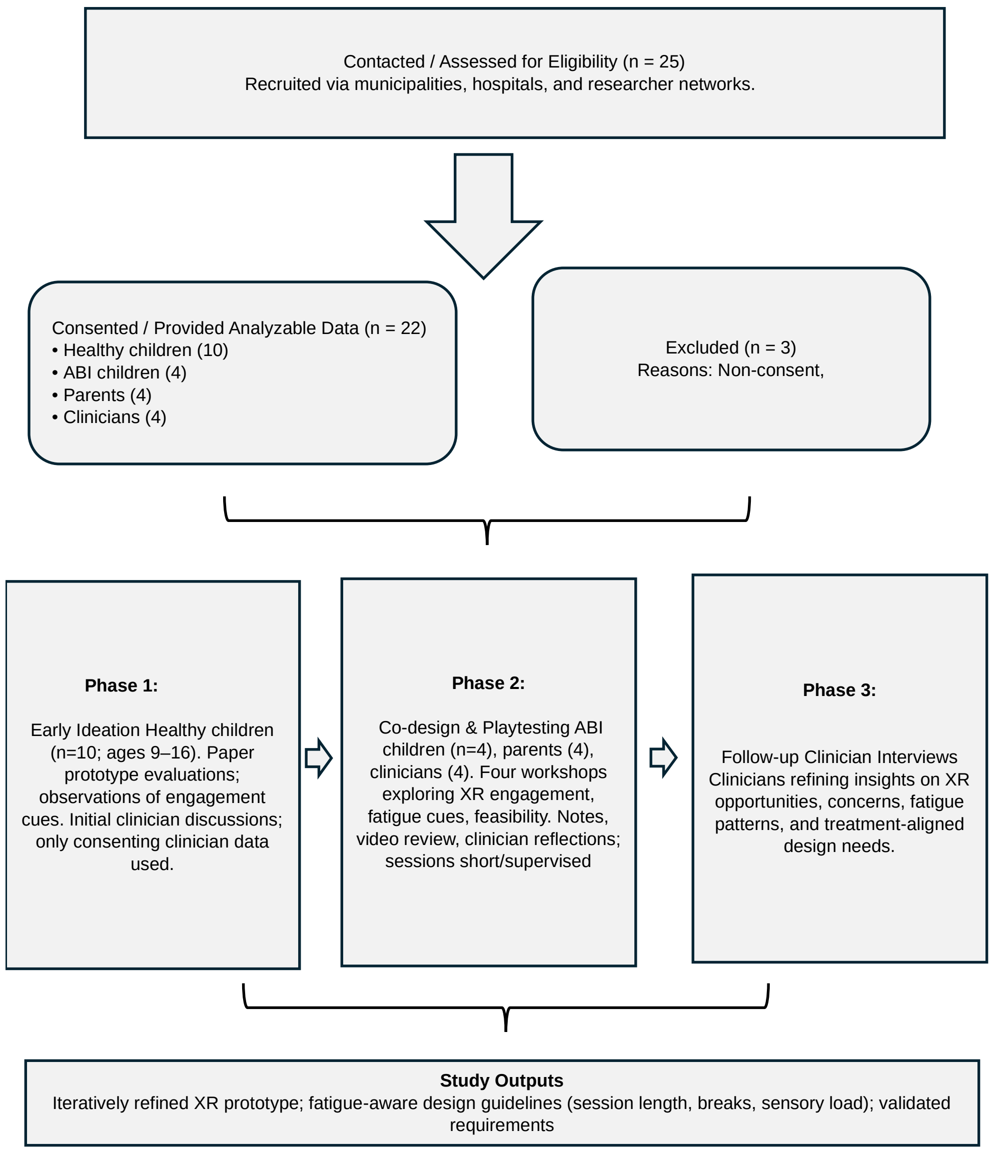
Contacted / Assessed for Eligibility (n = 25)
Recruited via municipalities, hospitals, and researcher networks.
Consented / Provided Analyzable Data (n = 22)
• Healthy children (10)
• ABI children (4)
• Parents (4)
• Clinicians (4)
Excluded (n = 3)
Reasons: Non-consent,
Phase 1:
Early Ideation Healthy children (n=10; ages 9–16). Paper prototype evaluations; observations of engagement cues. Initial clinician discussions; only consenting clinician data used.
Phase 2:
Co-design & Playtesting ABI children (n=4), parents (4), clinicians (4). Four workshops exploring XR engagement, fatigue cues, feasibility. Notes, video review, clinician reflections; sessions short/supervised
Phase 3:
Follow-up Clinician Interviews Clinicians refining insights on XR opportunities, concerns, fatigue patterns, and treatment-aligned design needs.
Study Outputs
Iteratively refined XR prototype; fatigue-aware design guidelines (session length, breaks, sensory load); validated requirements
